# Low Carotid Mean Flow Velocity: A Noninvasive Marker for Coronary Heart Disease—A Community-Based Study

**DOI:** 10.3390/diagnostics15081005

**Published:** 2025-04-15

**Authors:** Li-Chih Wu, Chao-Liang Chou, Shu-Hao Wu, Tzu-Wei Wu, Wei-Ren Lan, Chun-Fang Cheng, Shu-Xin Lu, Yih-Jer Wu, Li-Yu Wang

**Affiliations:** 1 Department of Cardiology, Hsinchu MacKay Memorial Hospital, Hsinchu City 30071, Taiwan; terminatorlw@gmail.com; 2 Division of Preventive Cardiology & Pulmonary Circulation Medicine, Department of Cardiovascular Medicine, MacKay Memorial Hospital, Taipei 10449, Taiwan; 3 Department of Medicine, MacKay Medical College, New Taipei 252005, Taiwan; chaoliangchou@gmail.com (C.-L.C.);; 4 Department of Neurology, MacKay Memorial Hospital, New Taipei 251020, Taiwan; z5x5c5v5b5@gmail.com; 5 Tamsui Health Station, Department of Health, New Taipei City Government, New Taipei 22001, Taiwan; aa3310@ntpc.gov.tw; 6 Institute of Biomedical Sciences, MacKay Medical College, New Taipei 252005, Taiwan

**Keywords:** carotid blood flow, resistant index, pulsatility index, coronary heart disease, hypertension, carotid Doppler ultrasonography

## Abstract

**Background:** Coronary heart disease (CHD) remains a leading cause of global mortality, often sharing pathophysiologic pathways with cerebrovascular atherosclerosis. Carotid duplex ultrasonography provides a convenient, noninvasive assessment of both vascular structure and hemodynamics. However, the clinical implications of specific carotid flow velocities for predicting CHD risk have not yet been fully explored. **Methods:** We conducted a case–control study using two community-based cohort datasets from Taiwan’s northern coastal region, enrolling individuals during two periods: from September 2010 to May 2011 and September 2014 to May 2020. Among 4102 participants aged 40–74 years, 45 were excluded for insufficient Doppler waveforms, leaving 4057 eligible subjects. Of these, 165 individuals with physician-confirmed myocardial infarction or prior coronary intervention/surgery were classified as CHD cases; the remaining 3892 served as controls. Carotid blood flow parameters—peak systolic (PSV), end-diastolic (EDV), and time-average maximal flow velocity (MFV), as well as resistance and pulsatility indices (RIs and PIs)—were determined by color Doppler ultrasound at the bilateral common carotid arteries (CCAs). Associations between these measurements and CHD were evaluated through logistic regression, adjusting for conventional cardiovascular risk factors. **Results:** Participants diagnosed with CHD exhibited significantly lower carotid flow velocities as well as higher RIs and PIs. After multivariable adjustments, right-sided common carotid artery (CCA) flow velocities have a relatively better predictive capacity than left-sided CCA flow velocities. However, left-sided RIs (adjusted OR per 0.1 increase = 1.41, *p* = 0.027) and PIs (adjusted OR per 1.0 increase = 1.60, *p* = 0.037) have better predictive value. Right-sided CCA MFV emerged as an independent predictor of CHD which was the most important (adjusted OR per 5.0 cm/s increase = 0.85; 95% CI: 0.77–0.95, *p* = 0.0038). As compared to subjects with high MFV (≥44.2 cm/s), the multivariable-adjusted OR of having CHD was significantly elevated for subjects with low MFV (<36.02 cm/s; OR = 1.79; 95% CI: 1.13–2.84, *p* = 0.013). Combinatory analysis further revealed that individuals with low right MFV, particularly when combined with hypertension, had substantially elevated odds of CHD. **Conclusions:** Reduced right CCA MFV may serve as a clinically informative signal for the presence of CHD. These findings highlight the potential role of detailed carotid Doppler profiling in refining CHD risk assessment, especially among hypertensive patients. Future prospective investigations are warranted to validate the predictive utility of the MFV for coronary events and to explore whether modifying this parameter through targeted interventions can mitigate cardiovascular risk.

## 1. Introduction

Atherosclerosis-related cardiovascular diseases (ASCVDs) are the leading cause of mortality worldwide, with coronary heart disease (CHD) accounting for approximately 16% of global deaths [1]. ASCVDs share common risk factors, including aging, cigarette smoking, diabetes mellitus, hypertension, and dyslipidemia, which contribute to the development and progression of vascular pathology among peripheral, cerebral, and coronary arteries. Given these shared pathophysiological mechanisms, it is not surprising that individuals with peripheral arterial disease [2] or ischemic stroke frequently exhibit coronary atherosclerosis, even in the absence of known CHD [3]. A comprehensive meta-analysis of 58 studies, encompassing 131,299 patients with a history of ischemic stroke or transient ischemic attack, found that the annual risk of myocardial infarction could be as high as 1.67% [4], underscoring the strong link between cerebrovascular and coronary atherosclerotic disease.

Carotid duplex ultrasonography has emerged as a powerful noninvasive tool for assessing carotid artery structure and hemodynamics. While previous studies have established a correlation between increased carotid intima-media thickness (IMT) or carotid plaques and elevated risk of CHD [5,6,7], the evidence of carotid flow velocity-related parameters in predicting CHD remains not fully explored. Low carotid end-diastolic velocity (EDV) has been independently linked to cardiovascular outcomes, but it is not a significant predictor for CHD [6]. Furthermore, it is not mentioned whether the EDV is from left- or right-sided carotid artery (CA), which have been reported to have a distinct flow pattern, IMT, and plaque distribution [5,8,9]. Although it has been reported that the peak systolic velocity (PSV) of the right internal CA is an independent predictor for angiographically significant coronary artery disease (CAD) even after adjusting IMT [5], the study population was confined to patients indicated for coronary angiography and so is highly selective, implying that the evidence for its use in the CHD screening in the general population might be not strong enough.

Despite these findings, there remains a lack of consensus regarding the clinical application of carotid flow velocity measurements in CHD risk stratification. This community-based case–control study aims to assess the relationship between detailed carotid Doppler-derived flow velocity parameters and the presence of CHD. The findings of this study could contribute to the development of improved risk assessment strategies for individuals at risk of CHD, ultimately aiding in early diagnosis and targeted intervention.

## 2. Methods

### 2.1. Study Subjects

**Figure 1:**
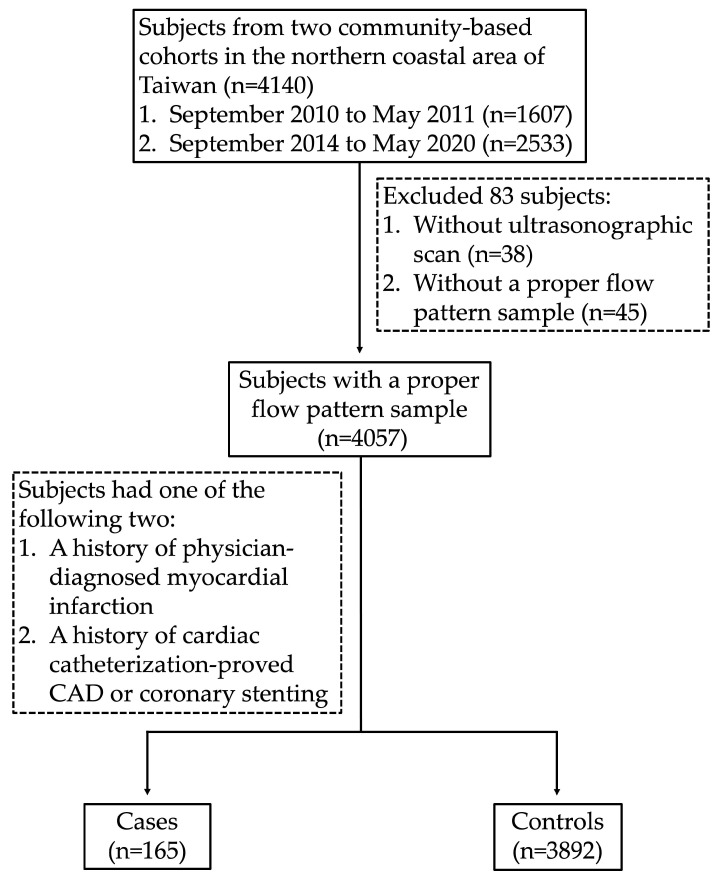
Consort flow diagram.

Participants in this study were recruited from two community-based cohort investigations targeting middle-aged and older adults residing in northern coastal Taiwan. The recruitment process took place in two phases: the first between September 2010 and May 2011 (period I) and the second between September 2014 and May 2020 (period II). A total of 4140 residents, aged 40 to 74 years, provided informed consent to participate in the study. Among these, 38 individuals who did not receive ultrasound scan were excluded. Additionally, another 45 individuals were excluded due to the failure to meet the criterion of possessing at least three consistent flow waveforms on Doppler ultrasound (Figure 1). The study protocol adhered to the ethical guidelines outlined in the 1975 Declaration of Helsinki and received approval from the institutional review boards of MacKay Medicine College (Approval No. P990001) and MacKay Memorial Hospital (Approval No. 14MMHIS075).

### 2.2. Anthropometric and Biochemical Measurements

Baseline anthropometric and clinical assessments followed standardized protocols as previously described [9]. Blood pressure was measured three times using a digital sphygmomanometer (UDEX-Twin; ELK Co., Daejon, Republic of Korea) following a 10-min seated rest in the morning. Each measurement was taken at least two minutes apart, and the mean of these readings was used for systolic (SBP) and diastolic (DBP) blood pressure analyses.

After an overnight fast of at least eight hours, venous blood samples were collected from each participant. Serum concentrations of total cholesterol, high-density lipoprotein cholesterol (HDL-C), low-density lipoprotein cholesterol (LDL-C), triglycerides, and fasting plasma glucose were determined using an automated analyzer (Toshiba TBA c16000; Toshiba Medical System, Holliston, MA, USA) with commercial reagent kits (Denka Seiken, Tokyo, Japan).

Coronary heart disease (CHD) was identified in individuals with a documented history of physician-diagnosed myocardial infarction or having received a bypass surgery or coronary stenting, or cardiac catheterization-proved CAD. Hypertension was defined as SBP ≥ 140 mmHg, DBP ≥ 90 mmHg, or the use of anti-hypertensive medication. Hyperlipidemia was diagnosed based on clinical criteria or lipid-lowering treatment. Diabetes mellitus (DM) was defined by a fasting plasma glucose ≥ 126 mg/dL or a history of insulin or oral hypoglycemic therapy. Current smoking status was classified as smoking at least four days per week in the past month, while current alcohol consumption was defined similarly based on drinking frequency.

### 2.3. Ultrasonographic Measurements of Carotid Blood Flow

**Figure 2:**
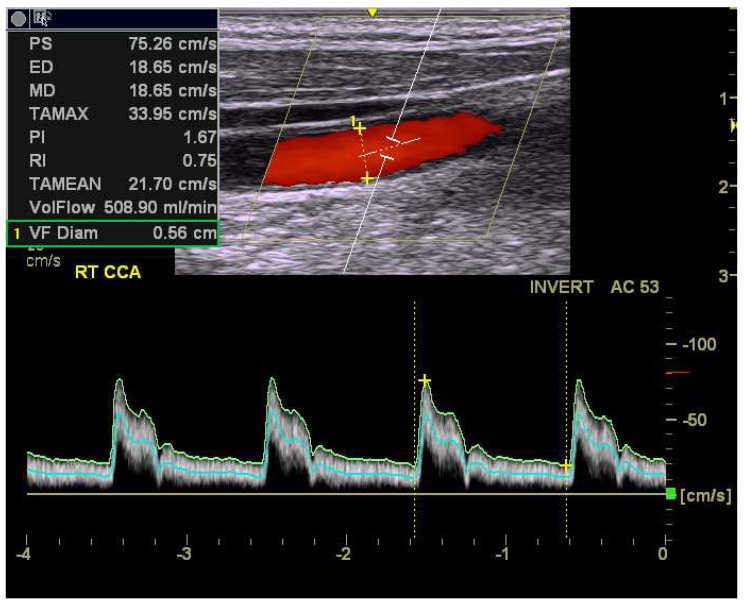
A representative Doppler image of the right CCA.

In this study, blood flow velocities, including peak systolic velocity (PSV), end-diastolic velocity (EDV), and time-average maximal flow velocity (MFV), in the middle segment of the common carotid artery (CCA) were assessed by color Doppler ultrasonography with PW-Doppler mode. In period I, one trained sonographer performed scans using the GE Healthcare ultrasound systems Vivid 7 or Vivid E9 (General Electric Company, Milwaukee, WI, USA). In period II, another sonographer, who was trained by the sonographer from period I, performed scans using the GE Healthcare ultrasound system Logiq E (General Electric Company, Milwaukee, WI, USA). The ultrasound systems were equipped with multi-frequency linear transducers L9-RS (3.33 to 10.0 MHz; General Electric Company, Milwaukee, WI, USA). The sonographers were blinded to participants’ clinical information when performing the ultrasound scans. Participants were positioned supine with their heads rotated approximately 45° away from the examined side. An insonation angle of ≤60° was maintained to ensure measurement accuracy, and the Doppler sample volume was set to encompass 0.5 to 2/3 of the arterial lumen. A valid blood flow assessment required at least three consecutive waveforms with consistent configuration (Figure 2). Additionally, pulsatility index (PI) and resistance index (RI) were automatically calculated using the following standard formulas:

$$\mathrm{PI}=\frac{\mathrm{PSV}-\mathrm{EDV}}{\mathrm{MFV}}\;\;\;\mathrm{RI}=\frac{\mathrm{PSV}-\mathrm{EDV}}{\mathrm{PSV}}$$

### 2.4. Statistical Analyses

Comparisons of anthropometric, biochemical, and carotid blood flow parameters between CHD and non-CHD groups were conducted using Student’s t and Chi-square tests. Variables demonstrating skewed distributions were log-transformed prior to analysis. The relationships between carotid flow indices and CHD were evaluated using odds ratios (ORs) with corresponding 95% confidence intervals (CIs). We used a four-step approach to assess the independent effects of blood flow velocities on CHD odds. First, cardio-metabolic risk factors showing significantly different between CHD cases and non-cases were subject to association analyses. Second, all significant cardio-metabolic risk factors were subject to multivariable logistic regression models with a stepwise selection method to obtain the best-fit model of conventional CVD risk factors. Age and sex were used in the multivariable analyses. The criteria for entering into and staying in the regression model for other cardio-metabolic risk factors were both set at 0.05. Third, each blood flow velocity was separately added to the best-fit model to assess their independent relationships with CHD. Finally, all significant blood flow velocities were included to the best-fit model with a stepwise selection method to obtain the most predictive model. All statistical analyses were performed using SAS 9.4 (SAS Institute Inc., Cary, NC, USA).

## 3. Results

### 3.1. Baseline Clinical Characteristics and Carotid Flow Parameters Between CHD and Non-CHD Subjects

Out of 4057 enrolled participants, 165 (4.1%) satisfied the criteria for CHD and served as cases. Table 1 summarizes the anthropometric and biochemical measurements stratified by CHD status. Individuals with CHD had significantly higher mean values of age, body mass index (BMI), waist circumference, and waist-to-hip ratio, as well as greater proportions of male sex, hypertension, hyperlipidemia, DM, and cigarette smoking, compared with the non-CHD controls. In contrast, total cholesterol, LDL-C, and HDL-C levels were lower among the CHD group, reflecting higher baseline lipid therapy for secondary prevention. Other measurements showed no significant differences between the two groups. Table 2 presents a comparison of carotid hemodynamic parameters between participants with and without CHD. Carotid flow velocities (PSV, EDV, and MFV) were uniformly lower in the CHD group, while both PI and RI were significantly higher relative to controls.

### 3.2. Univariate and Multivariate Analyses for the Carotid Flow Parameters in Predicting the Presence of CHD

Univariable analyses (Table 3) indicated that increased carotid flow velocities were inversely correlated with CHD. Specifically, each 5.0 cm/s increment in PSV corresponded to ORs for CHD ranging from 0.91 to 0.93. Comparable increases in EDV and MFV yielded ORs of 0.62 to 0.69 and 0.73 to 0.79, respectively. By contrast, higher PI and RI were positively associated with CHD. Each 0.1-unit rise in RI correlated with ORs of 1.94 to 2.23, while each 1.0-unit increase in PI was linked to ORs of 2.14 to 2.64. Table 3 also details the multivariable logistic regression findings. After controlling for the effects of conventional cardiovascular risk factors, including age, sex, cigarette smoking, hyperlipidemia, SBP, and low-density lipoprotein (LDL) levels, EDV remained significantly predictive of CHD, while PSV became non-significant. The adjusted ORs for average MFV and right-sided MFV attained statistical significance and left-sided MFV reached borderline significance. Elevated left and mean RI and PI were similarly tied to increased CHD risk, with right RI and PI displaying only borderline significance in the fully adjusted models.

A stepwise regression approach incorporating all significant carotid flow variables revealed that the right MFV was the most robust independent predictor of CHD. Each additional 5.0 cm/s in the right MFV was associated with an adjusted OR of 0.85 (95% CI: 0.77–0.95) (Table 3). Models combining traditional cardiovascular risk factors with either right EDV or average MFV demonstrated similar discriminative ability (both c-statistics = 0.754), with adjusted ORs of 0.82 (95% CI: 0.70–0.95) and 0.81 (95% CI: 0.68–0.95), respectively. To further explore the predictability of CCA MFV and EDV, a multivariable model which contained traditional cardiovascular risk factors and CCA MFV and EDV was generated. The multivariable-adjusted ORs for per 5 cm/s increase in CCA MFV and EDV were 0.85 (95% CI, 0.68–1.06) and 0.99 (95% CI, 0.71–1.37), respectively. The results indicated that in the presence of CCA MFV, the effect of CCA EDV was nearly negligible.

### 3.3. Right CCA MFV and Hypertension Jointly Predict the Presence of CHD

For practical applications, right CCA MFV was divided into three categories, low (<36.0 cm/s), medium (36.0–44.1 cm/s), and high (≥44.2 cm/s), as depicted in Table 4. The prevalence of CHD in these groups was 7.1%, 2.9%, and 2.2%, respectively. Compared with the high MFV category, the adjusted OR of CHD was 1.79 (95% CI: 1.13–2.84) in the low MFV group (model 1), whereas the medium MFV group showed only a modest elevation in CHD risk. In model 2, the highest prevalence of CHD was observed among hypertensive individuals with low right MFV. Relative to normotensive subjects in the high MFV group, hypertensive individuals with low right MFV had an adjusted OR of 4.79 (95% CI: 2.63–8.72). Hypertensive participants with medium or high right MFV also demonstrated significantly increased ORs compared with those who were normotensive and had high MFV.

## 4. Discussion

In the present study, we sought to identify noninvasive hemodynamic parameters obtained via carotid Doppler ultrasound that could be linked to the risk of coronary heart disease (CHD). To our knowledge, this is the first observational study to specifically evaluate carotid MFV as a potential indicator of CHD. Our findings demonstrate that lower MFV in the right CCA is associated with a higher likelihood of CHD, and this relationship appears to be particularly pronounced in individuals with hypertension. This observation underscores the intricate interplay between vascular hemodynamics and systemic cardiovascular risk factors, suggesting that an integrated assessment of carotid flow parameters might better elucidate the pathophysiological continuum of atherosclerosis.

Previous investigations have emphasized the predictive value of PSV or EDV for future cardiovascular events, but these studies have their limitations. In a cross-sectional study, Zhang et al. noted that patients exhibiting both increased PSV of the right internal carotid artery (ICA) and elevated carotid plaque scores had a heightened risk of CAD [5]. However, this study enrolled only patients indicated for coronary angiography (representing a higher-risk population), and therefore, the results should be generalized to the general population with caution. Meanwhile, Chuang et al. found that not only PSV but also EDV in the common carotid artery independently predicted cardiovascular events [6], which aligned with similar findings in a study on the Korean population showing that multiple flow parameters (e.g., average CCA PSV and EDV) were independently associated with cardiovascular disease even after adjusting for traditional risk factors, IMT, and carotid plaques [10]. However, the identified carotid flow parameters are either insignificant predictors for CHD [6] or not specifically demonstrated to be CHD-associated [10]. Lee et al., in a small-scale study, demonstrated that RI is significantly associated with CHD risk scores in patients with hypertension but did not reveal the association with other carotid flow parameters [11]. Nevertheless, these studies collectively still underscore the importance of examining several carotid flow indices to capture a more comprehensive view of vascular pathology. Moreover, emerging evidence suggests that incorporating diverse hemodynamic parameters and risk factor profiles can enhance the detection of subclinical carotid atherosclerosis, thereby refining risk stratification for CHD [11].

The possible pathophysiological underpinnings linking reduced carotid blood flow to CHD may involve both systemic atherosclerosis and compromised left ventricular function. Atherosclerosis is known to affect multiple vascular beds simultaneously, and it shares common risk factors—such as hypertension, dyslipidemia, and diabetes mellitus—that contribute to its progression in both the carotid and coronary territories [2,12]. In line with this notion, our study also revealed that left CCA RIs and PIs, indicators of atherosclerosis and vascular stiffness [13], were also good predictors for the presence of CHD after multivariable adjustment. The close link is further supported by the reports showing that RI and/or PI are independently correlated with ASCVD risk scores [11,14] and CAD [5]. Inflammatory mediators, including interleukin-6 and high-sensitivity C-reactive protein, play a central role in atherosclerosis and have been shown to correlate with carotid hemodynamic parameters [15]. Notably, diabetes mellitus itself has been associated with alterations in carotid flow parameters [12], potentially exacerbating the atherosclerotic burden and intensifying cardiovascular risk. In the present study, we identified that hypertension history (but not SBP) and right CCA MFV are independently and jointly predictive of the presence of CHD. This phenomenon should be, however, explained with caution. It may either reflect a synergistic risk of combining low right CCA MFV and hypertension on CHD development [11], or just reflect a confounding background with a high percentage of using β-blockers, angiotensin-converting enzyme inhibitors, or angiotensin receptor antagonists, which are traditionally recommended in patients with confirmed CHD [16].

Furthermore, reduced left ventricular stroke volume or overt heart failure, which is the final pathway of CHD, also lead to diminish overall cerebral perfusion and may be intricately tied to diminished carotid flow velocities [17]. This confluence of hemodynamic and inflammatory processes highlights the complex nature of CHD and its close relationship with carotid vascular indices.

The importance of measuring MFV, in addition to peak systolic and end-diastolic velocities, lies in its potential to integrate various phases of the cardiac cycle [14]. By capturing both systolic and diastolic elements, mean velocity may reflect a broader spectrum of vascular and cardiac interactions, especially among individuals burdened by multiple comorbidities. Given that risk assessment tools for atherosclerosis and cardiovascular disease continue to evolve, future studies may benefit from integrating MFV into existing risk scores to evaluate its additive prognostic value.

The right CCA MFV was found to be the independent and most robust predictor for the presence of CHD. It has been reported in a relatively large-scale study that left- and right-sided carotid flow parameters are not always identical [8]. Contrary to our findings, Zhang et al. have demonstrated that the increased right ICA PSV, but not left ICA PSV, is a significant predictor for the presence of significant CAD [5]. This contradictory phenomenon may be confounded by local stenosis results from carotid plaques, which are the strongest factor associated with angiographic CAD in that study. Interestingly, our previous study has contrarily shown that left CAs are more likely to form plaques than right CAs [9], and together with the evidence showing that carotid flow parameters are independent of carotid plaque severities to predict cardiovascular events [5,10] imply that the flow pattern may be more sensitive and predictive in the relatively plaque-free right CA. This speculation was support by this study. The prevalence rates of carotid plaque were 60.0% and 34.5% in CHD cases and non-cases (*p* < 0.0001), respectively. Additionally, the multivariable-adjusted ORs of having CHD per 5.0 cm/s MFV increase in the model including the presence of carotid plaque were nearly un-changed (OR = 0.86; 95% CI: 0.77~0.94; *p* = 0.0022). In line with this notion, carotid flow parameters are also independent of IMT in the prediction of cardiovascular events [6,10,18].

There are several limitations to our study. First, CHD was defined according to a documented history of myocardial infarction or coronary intervention/surgery, rather than more direct assessments of disease severity (e.g., echocardiography, treadmill testing, myocardial perfusion imaging, or coronary angiography) [16]. Consequently, we could not capture detailed gradations of CHD severity or disease activity. Second, the study population was derived from community-based data in Taiwan, and the generalizability of our findings to other ethnicities remains uncertain. Larger, multiethnic, population-based cohorts are warranted to validate the external applicability of these results. Third, the cross-sectional nature of this case–control design inherently limits causal inferences. Prospective, well-controlled longitudinal studies are essential to confirm our findings and further clarify the prognostic implications of carotid mean flow velocity. Future investigations should also consider incorporating genetic markers associated with coronary, carotid atherosclerosis, and cardiovascular risk factors [12], as these might offer a more comprehensive perspective on the interplay between carotid hemodynamics and systemic cardiometabolic risk.

## 5. Conclusions

In summary, our study adds to the growing body of evidence that noninvasive parameters derived from carotid Doppler ultrasound may be key indicators of CHD. Specifically, our findings on lower right CCA MFV underscore the value of assessing multiple carotid flow indices—beyond peak systolic velocity—to better predict CHD. This approach may ultimately enhance awareness of the potential presence of CHD and guide more targeted interventions for individuals at heightened risk of CHD.

## Figures and Tables

**Table 1:** Baseline clinical characteristics of CHD patients and non-CHD controls.

Variables	Non-CHD Controls (*n* = 3892)		CHD Patients (*n* = 165)		*p*-Value
**Continuous variables**	**Mean**	**SD**	**Mean**	**SD**	
Age (years)	56.0	8.9	61.3	8.5	<0.0001
Body mass index (kg/m^2^)	24.6	3.6	25.2	3.7	0.041
Waist circumference (cm)	85.6	10.1	88.6	9.8	0.0002
HIP (cm)	96.5	7.2	96.9	6.7	0.44
Waist-to-hip ratio (%)	88.6	7.1	91.3	6.4	<0.0001
SBP (mmHg)	126.5	18.7	127.6	17.1	0.46
DBP (mmHg)	76.3	12.6	75.5	11.4	0.38
Total cholesterol (mg/dL)	204.8	38.6	184.1	39.3	<0.0001
LDL (mg/dL)	121.4	32.5	104.9	31.5	<0.0001
HDL (mg/dL)	55.7	15.0	51.4	15.5	0.0004
Log (Trigelyceride)	4.6	0.6	4.7	0.6	0.11
**Categorical variables**	***n***	**%**	***n***	**%**	
Female	2523	64.8	86	52.1	0.0008
Hypertension	922	23.7	93	56.4	<0.0001
Hyperlipidemia	1124	28.9	82	49.7	<0.0001
Diabetes mellitus	417	10.7	39	23.6	<0.0001
Cigarette smoking	827	21.3	52	31.5	0.0019
Alcohol drinking	498	12.8	28	17.0	0.12

CHD, coronary heart disease; HIP, hip circumference; SBP, systolic blood pressure; DBP, diastolic blood pressure; LDL, low-density lipoprotein; HDL, high-density lipoprotein.

**Table 2:** Comparisons of carotid blood flows, resistance index, and pulsatility index between CHD patients and non-CHD controls.

Variables	Non-CHD Controls (*n* = 3892)		CHD Patients (*n* = 165)		*p*-Value
**Right CCA**	**Mean**	**SD**	**Mean**	**SD**	
PSV (cm/s)	86.2	20.2	79.6	19.4	<0.0001
EDV (cm/s)	23.4	6.4	20.1	6.4	<0.0001
MFV (cm/s)	40.7	9.2	35.8	9.2	<0.0001
RI	0.73	0.06	0.75	0.06	<0.0001
PI	1.57	0.36	1.70	0.39	<0.0001
**Left CCA**					
PSV (cm/s)	85.6	19.0	80.9	19.0	0.002
EDV (cm/s)	24.5	6.5	21.6	7.0	<0.0001
MFV (cm/s)	41.6	9.2	37.8	9.7	<0.0001
RI	0.71	0.06	0.73	0.06	<0.0001
PI	1.49	0.34	1.61	0.37	<0.0001
**Average of right and left CCA**					
PSV (cm/s)	85.9	18.3	80.2	18.2	0.0001
EDV (cm/s)	23.9	5.9	20.8	6.3	<0.0001
MFV (cm/s)	41.1	8.5	36.8	8.9	<0.0001
RI	0.71	0.06	0.73	0.06	<0.0001
PI	1.53	0.32	1.66	0.35	<0.0001

CHD, coronary heart disease; CCA, common carotid artery; PSV: peak systolic velocity; EDV, end-diastolic velocity; MFV, time-average maximal flow velocity; RI, resistance index; PI, pulsatility index.

**Table 3:** Association analyses for having CHD with carotid blood flows, resistance index, and pulsatility index.

	Univariable			Multivariable Adjusted ^1^		
	OR	(95% CI)	*p*	OR	(95% CI)	*p*
**Right CCA**						
PSV (per 5 cm/s)	0.92	(0.88–0.95)	<0.0001	0.96	(0.92–1.01)	0.094
EDV (per 5 cm/s)	0.64	(0.56–0.73)	<0.0001	0.82	(0.70–0.95)	0.011
MFV (per 5 cm/s)	0.73	(0.67–0.80)	<0.0001	0.85	(0.77–0.95)	0.0038
RI (per 0.1)	1.94	(1.46–2.57)	<0.0001	1.32	(0.97–1.78)	0.073
PI (per 1.0)	2.22	(1.55–3.19)	<0.0001	1.50	(0.98–2.29)	0.065
**Left CCA**						
PSV (per 5 cm/s)	0.93	(0.90–0.98)	0.0019	0.98	(0.93–1.02)	0.35
EDV (per 5 cm/s)	0.69	(0.60–0.78)	<0.0001	0.86	(0.74–0.99)	0.037
MFV (per 5 cm/s)	0.79	(0.72–0.86)	<0.0001	0.91	(0.82–1.01)	0.069
RI (per 0.1)	2.01	(1.53–2.65)	<0.0001	1.41	(1.04–1.90)	0.027
PI (per 1.0)	2.14	(1.49–3.06)	<0.0001	1.60	(1.03–2.50)	0.037
**Average of right and left CCA**						
PSV (per 5 cm/s)	0.91	(0.87–0.96)	<0.0001	0.97	(0.92–1.01)	0.15
EDV (per 5 cm/s)	0.62	(0.54–0.71)	<0.0001	0.81	(0.68–0.95)	0.011
MFV (per 5 cm/s)	0.73	(0.66–0.80)	<0.0001	0.86	(0.77–0.97)	0.0098
RI (per 0.1)	2.23	(1.65–3.01)	<0.0001	1.46	(1.04–2.04)	0.027
PI (per 1.0)	2.64	(1.73–3.97)	<0.0001	1.72	(1.05–2.83)	0.032

^1^ Adjusted for age, sex, cigarette smoking, hypertension, hyperlipidemia, SBP, and LDL. CHD, coronary heart disease; CCA, common carotid artery; PSV, peak systolic velocity; EDV, end-diastolic velocity; MFV, time-average maximal flow velocity; RI, resistance index; PI, pulsatility index.

**Table 4:** Multivariable analyses for having CHD with MFV of right CCA and hypertension.

			CHD Cases		Model 1 ^1^			Model 2 ^1^		
		Total	No.	%	OR	(95% CI)	*p*	OR	(95% CI)	*p*
**Hypertension**										
No		3042	72	2.4	1.00			-		
Yes		1015	93	9.2	2.84	(1.95–4.13)	<0.0001	-		
**Right CCA MFV (cm/s)**										
≥44.2		1350	30	2.2	1.00			-		
36.0~44.1		1365	40	2.9	1.06	(0.65–1.74)	0.81	-		
<36.0		1342	95	7.1	1.79	(1.13–2.84)	0.013	-		
**Hypertension**	**MFV (cm/s)**									
No	≥44.2	1152	20	1.7	-			1.00		
No	36.0~44.1	1061	23	2.2	-			1.13	(0.61–2.08)	0.71
No	<36.0	829	29	3.5	-			1.63	(0.89–2.98)	0.11
Yes	≥44.2	198	10	5.1	-			2.47	(1.10–5.56)	0.029
Yes	36.0~44.1	304	17	5.6	-			2.46	(1.20–5.03)	0.014
Yes	<36.0	513	66	12.9	-			4.79	(2.63–8.72)	<0.0001

^1^ Adjusted for age, sex, cigarette smoking, hyperlipidemia, SBP, and LDL-C. CHD, coronary heart disease; CCA, common carotid artery; MFV, time-average maximal flow velocity.

## Data Availability

The data underlying this article are available in this article and can be obtained from authors upon reasonable request.

## Abbreviations

The following abbreviations are used in this article:

ASCVDs	Atherosclerosis-related cardiovascular diseases
CHD	Coronary heart disease
IMT	Intima-media thickness
CA	Carotid artery
CCA	Common carotid artery
ICA	Internal carotid artery
MFV	Mean flow velocity
PSV	Peak flow velocity
EDV	End diastolic flow velocity
PI	Pulsatility index
RI	Resistance index
CAD	Coronary artery disease
LDL-C	Low-density lipoprotein cholesterol
HDL-C	High-density lipoprotein cholesterol
SBP	Systolic blood pressure
DBP	Diastolic blood pressure

## References

[1] Song P., Fang Z., Wang H., Cai Y., Rahimi K., Zhu Y., Fowkes F.G.R., Fowkes F.J.I., Rudan I. (2020). Global and regional prevalence, burden, and risk factors for carotid atherosclerosis: A systematic review, meta-analysis, and modelling study. Lancet Glob. Health.

[2] Satiroglu O., Kocaman S.A., Karadag Z., Temiz A., Cetin M., Canga A., Erdogan T., Bostan M., Cicek Y., Durakoglugil E. (2012). Relationship of the angiographic extent of peripheral arterial disease with coronary artery involvement. J. Pak. Med. Assoc..

[3] Amarenco P., Lavallée P.C., Labreuche J., Ducrocq G., Juliard J.-M., Feldman L., Cabrejo L., Meseguer E., Guidoux C., Adraï V. (2011). Prevalence of coronary atherosclerosis in patients with cerebral infarction. Stroke.

[4] Boulanger M., Béjot Y., Rothwell P.M., Touzé E. (2018). Long-term risk of myocardial infarction compared to recurrent stroke after transient ischemic attack and ischemic stroke: Systematic review and meta-analysis. J. Am. Heart Assoc..

[5] Zhang H., Liu M., Ren T., Wang X., Liu D., Xu M., Han L., Wu Z., Li H., Zhu Y. (2015). Associations between carotid artery plaque ccore, carotid hemodynamics and coronary heart disease. Int. J. Environ. Res. Public Health.

[6] Chuang S.-Y., Bai C.-H., Cheng H.-M., Chen J.-R., Yeh W.-T., Hsu P.-F., Liu W.-L., Pan W.-H. (2016). Common carotid artery end-diastolic velocity is independently associated with future cardiovascular events. Eur. J. Prev. Cardiol..

[7] Yu J.B., Wang X.L., An Z.J., Zhu D.L., Xu L., Xu T., Wang D., Qu Y., Li N., Li L.H. (2023). Predicting coronary artery disease by carotid color doppler ultrasonography. Eur. Rev. Med. Pharmacol. Sci..

[8] Zócalo Y., Bia D. (2021). Sex- and age-related physiological profiles for brachial, vertebral, carotid, and femoral arteries blood flow velocity parameters during growth and aging (4–76 years): Comparison with clinical cut-off levels. Front. Physiol..

[9] Chou C.-L., Wu Y.-J., Hung C.-L., Liu C.-C., Wang S.-D., Wu T.-W., Wang L.-Y., Yeh H.-I. (2018). Segment-specific prevalence of carotid artery plaque and stenosis in middle-aged adults and elders in Taiwan: A community-based study. J. Formos. Med. Assoc..

[10] Chung H., Jung Y.H., Kim K.H., Kim J.Y., Min P.K., Yoon Y.W., Lee B.K., Hong B.K., Rim S.J., Kwon H.M. (2016). Carotid artery end-diastolic velocity and future cerebro-cardiovascular events in asymptomatic high risk patients. Korean Circ. J..

[11] Lee M.Y., Wu C.M., Chu C.S., Lee K.T., Sheu S.H., Lai W.T. (2008). Association of carotid hemodynamics with risk of coronary heart disease in a Taiwanese population with essential hypertension. Am. J. Hypertens..

[12] Wu T.W., Wu Y.J., Chou C.L., Cheng C.F., Lu S.X., Wang L.Y. (2024). Hemodynamic parameters and diabetes mellitus in community-dwelling middle-aged adults and elders: A community-based study. Sci. Rep..

[13] Baran J., Kleczyński P., Niewiara Ł., Podolec J., Badacz R., Gackowski A., Pieniążek P., Legutko J., Żmudka K., Przewłocki T. (2021). Importance of increased arterial resistance in risk prediction in patients with cardiovascular risk factors and degenerative aortic stenosis. J. Clin. Med..

[14] Lu Y.-C., Chen P.-J., Lu S.-N., Liang F.-W., Chuang H.-Y. (2024). Comparing carotid artery velocities with current ASCVD risk stratification: A novel approach to simpler risk assessment. J. Epidemiol. Glob. Health.

[15] Manabe S., Okura T., Watanabe S., Higaki J. (2005). Association between carotid haemodynamics and inflammation in patients with essential hypertension. J. Hum. Hypertens..

[16] Ueng K.C., Chiang C.E., Chao T.H., Wu Y.W., Lee W.L., Li Y.H., Ting K.H., Su C.H., Lin H.J., Su T.C. (2023). 2023 Guidelines of the Taiwan Society of Cardiology on the Diagnosis and Management of Chronic Coronary Syndrome. Acta Cardiol. Sin..

[17] Cheong I., Otero Castro V., Sosa F.A., Tort Oribe B., Merlo P.M., Tamagnone F.M. (2023). Carotid flow as a surrogate of the left ventricular stroke volume. J. Clin. Monit. Comput..

[18] Chuang S.-Y., Bai C.-H., Chen J.-R., Yeh W.-T., Chen H.-J., Chiu H.-C., Shiu R.-S., Pan W.-H. (2011). Common carotid end-diastolic velocity and intima-media thickness jointly predict ischemic stroke in Taiwan. Stroke.

